# An automatic method using MFCC features for sleep stage classification

**DOI:** 10.1186/s40708-024-00219-w

**Published:** 2024-02-10

**Authors:** Wei Pei, Yan Li, Peng Wen, Fuwen Yang, Xiaopeng Ji

**Affiliations:** 1 School of Mathematics, Physics and Computing, University of Southern Queensland, Toowoomba, QLD 4350 Australia; 2 School of Engineering, University of Southern Queensland, Toowoomba, QLD 4350 Australia; 3 School of Engineering and Built Environment, Griffith University, Gold Coast, QLD 4222 Australia

**Keywords:** Sleep stages, Convolutional neural network, Mel-frequency cepstral coefficients, Long short-term memory

## Abstract

Sleep stage classification is a necessary step for diagnosing sleep disorders. Generally, experts use traditional methods based on every 30 seconds (s) of the biological signals, such as electrooculograms (EOGs), electrocardiograms (ECGs), electromyograms (EMGs), and electroencephalograms (EEGs), to classify sleep stages. Recently, various state-of-the-art approaches based on a deep learning model have been demonstrated to have efficient and accurate outcomes in sleep stage classification. In this paper, a novel deep convolutional neural network (CNN) combined with a long short-time memory (LSTM) model is proposed for sleep scoring tasks. A key frequency domain feature named Mel-frequency Cepstral Coefficient (MFCC) is extracted from EEG and EMG signals. The proposed method can learn features from frequency domains on different bio-signal channels. It firstly extracts the MFCC features from multi-channel signals, and then inputs them to several convolutional layers and an LSTM layer. Secondly, the learned representations are fed to a fully connected layer and a softmax classifier for sleep stage classification. The experiments are conducted on two widely used sleep datasets, Sleep Heart Health Study (SHHS) and Vincent’s University Hospital/University College Dublin Sleep Apnoea (UCDDB) to test the effectiveness of the method. The results of this study indicate that the model can perform well in the classification of sleep stages using the features of the 2-dimensional (2D) MFCC feature. The advantage of using the feature is that it can be used to input a two-dimensional data stream, which can be used to retain information about each sleep stage. Using 2D data streams can reduce the time it takes to retrieve the data from the one-dimensional stream. Another advantage of this method is that it eliminates the need for deep layers, which can help improve the performance of the model. For instance, by reducing the number of layers, our seven layers of the model structure takes around 400 s to train and test 100 subjects in the SHHS1 dataset. Its best accuracy and Cohen’s kappa are 82.35% and 0.75 for the SHHS dataset, and 73.07% and 0.63 for the UCDDB dataset, respectively.

## Introduction

Sleep quality is one of the most critical health indicators. Poor sleep quality affects people’s daily lives and causes psychological issues, narcolepsy, and insomnia [1]. Most sleep issues and diseases are highly correlated with the period of each sleep stage phase [2]. Sleep stage classification helps to diagnose the physiological status of various diseases, such as stroke and cerebrovascular diseases.

For sleep stage classification, researchers usually use the sleep guidelines of the American Academy of Sleep Medicine (AASM) [3] and Rechtschaffen and Kales (R&K) [4]. The sleep stages are divided into six stages in R&K rules, referring to awake (W), rapid eye movement (REM), and stages 1–4 (S1, S2, S3, and S4). According to the AASM, experts separate sleep states into five stages as the AASM standard merges S3 and S4 into S3 as one stage. Among them, S1 and S2 are called light sleep, and S3 is called slow-wave sleep [5]. Diagnosis of sleep stage classification and sleep disorders often rely on the collected polysomnography (PSG) recordings. PSG recordings normally include electrocardiogram (ECG), electroencephalogram (EEG), electromyography (EMG), electrooculogram (EOG), respiration, oxygen saturation, and airflow. Most certified experts perform their PSG records and sleep staging signals in PSG at 30-second intervals [6].

Manual sleep stage classification is time-consuming and subject to experts’ experience and knowledge [7–9]. To improve the efficiency of sleep scoring and subsequently relieved the constraints of the workforce, several automatic sleep stage classification methods have been reported [10–23]. These include time-domain statistics method [10], frequency-domain analysis [11], time-frequency analysis [12], and graph domain analysis [13]. The performance of these approaches was reported to be different [13, 14]. For example, Zhu et al. [13] used graph domain features from EEG recordings and classified them using a support vector machine (SVM) into 2–6 stages. They reported a kappa coefficient of 81% and a classification accuracy of 87.5%. Diykh et al. [14] proposed a least square SVM (LS-SVM) model that was used for each extracted statistical feature from each EEG epoch. That method achieved an average accuracy of 96.74% based on the AASM standard and an accuracy of 96% according to the R&K standard. Existing research [10–14] have proved that using machine learning methods with handcrafted features for automatic sleep scoring is effective. However, the results of machine learning approaches strongly rely on the types of handcrafted features and dataset-dependant. Therefore, the performance and suitability of a trained machine learning model are likely to be dependent on the given datasets.

Several researchers reported some good sleep stage classification results using deep learning-based methods. Deep learning models have become popular in EEG research as they can automatically extract key features and produce outstanding performances. In the following deep learning algorithms [15–23], the authors proposed different novel models based on the sleep bio-signals to classify the sleep stages. In several methods [15–19], the features were extracted by a convolutional neural network (CNN). The methods [20–23] used several complex and efficient deep neural networks for sleep stage classification. Huy et al. [15] extracted time–frequency features from EEG, EMG, and EOG signals using a CNN model and they achieved an accuracy of 83%. Kuo et al. [16] proposed a method that combined a CNN and a decision-visualization technique in their study. They used a continuous wavelet transform (CWT) and resized the image to fit the shape of the network that pre-processed each training epoch. They applied a smoothing rule and trained a deep learning model that produced an accuracy, F1-score, and Cohen’s kappa of 93.78%, 0.91, and 0.88, respectively. Pei et al. [17] designed a novel hybrid deep learning model based on a CNN and a gated recurrent unit (GRU) that classified sleep stages from multi-channel biological signals. They reported an accuracy of 83.15% and a Cohen’s kappa of 76%. Their results showed that the performance of the CNN with different domain features was excellent. The authors, however, also reported that they added an extra data pre-processing step before training the CNN. Tripathy and Acharya [18] proposed a deep neural network (DNN) model that classified RR-time series, which is the time interphase between two successive R-waves on the ECG, from ECG signals for sleep stage classification. They reported that the framework produced an 85.51% accuracy for the sleep stage classification. Ji et al. [19] used different types of multi-channel EEG, EMG, EOG, and ECG data to train a novel deep learning model named jumping knowledge spatial–temporal graph convolutional network (JK-STGCN). Their model extracted spatial features from multi-channels and sleep stage’s transition characteristics. The authors reported an overall accuracy of 83.1%, an F1-score of 0.814, and a Cohen’s kappa of 0.782. This study demonstrates that other types of deep neural networks can perform well as the CNN-based models. Qu et al. [20] designed a deep multi-scale architecture based on different EEG frequency bands for sleep stage classification. Their method produced good results and was more efficient. Supratak and Guo [21] developed a deep learning model named TinySleepNet. They reported that the TinySleepNet had a better performance than the state-of-the-art methods with the same parameters and model architecture. Jia et al. [22] proposed a SleepPrintNet model to classify the sleep stages in time series. SleepPrintNet includes two EOG and EMG feature extraction modules and one EEG spectral–spatial feature extraction module. SleepPrintNet was the first attempt to combine multi-model simultaneously and learn the EEG spectral–spatial information by the deep learning model for sleep stage classification. The spectral–spatial features represent the same frequency band of each epoch; they combined the location of each electrode and get a 2D matrix. Phan et al. [23] designed a novel deep learning architecture based on a sequence-to-sequence sleep staging model and transformer framework named SleepTransformer. Most of the state-of-the-art deep learning algorithms can have an excellent performance. The main reason is that model powerful computers have the capacity to support the complex and deeper learning frameworks. This study proposes the application of an efficient CNN model with a long short-term memory (LSTM) to extract the Mel-frequency Cepstral Coefficient (MFCC) features for sleep stage classification.

The rest of the paper is organized as follows: in Sect. 2, the MFCC method and the deep learning architecture are introduced as the background for the following sections, and the experimental data are also briefly explained in Sect. 2. Section 3 presents the results of the proposed method and compares them with other state-of-the-art methods. Finally, the conclusions are given in Sect. 4.

## Materials and methodology

### Experimental data

To demonstrate the generality of the proposed method, the classification experiments are conducted based on two public datasets: Sleep Heart Health Study (SHHS) dataset [24] and Vincent’s University Hospital/University College Dublin Sleep Apnoea (UCDDB) dataset [25]. The SHHS dataset is a multi-center cohort study, with aim of assessing the result of sleep-disordered breathing and two datasets are included, namely the SHHS1 dataset and the SHHS2 dataset. Each PSG in SHHS1 dataset was chosen with one EMG signal with a sample rate of 125 Hz, and two EEG signals (C4A1 and C3A2) with a sample rate of 125 Hz. Similarly, the same channel is selected from the UCDDB dataset. The UCDDB database collected 25 overnight PSG recordings. It has a different sample rate from the SHHS1 dataset. The EMG signal has a sample rate of 64 Hz, and two EEG signals (C4A1 and C3A2) have a sample rate of 128 Hz. For these two datasets, we considered five sleep stages: wake, REM, stage 1, stage 2, and stage 3 (combined stage 3 and stage 4). Table 1 presents the detailed distribution of sleep stages from the SHHS1 dataset and the UCDDB dataset.

**Table 1:** The information of two datasets

Dataset	Wake	REM	Stage 1	Stage 2	Stage 3	Total
UCDDB	4707	3016	3403	6985	2663	20774
SHHS1-100	32881	14792	3683	40243	11841	103440

### Mel-frequency cepstral coefficients

The extraction of signal representative features is essential in sleep stage classification. Human ears are unable to hear and identify the biological signals, like EEG, or ECG, as those signal frequencies and amplitudes are out of the range of people’s capacity. The goal of the MFCC feature is to simulate the perception of signals by humans using a more discriminative approach. It achieves this by transforming the non-linear Mel scale’s energy spectrum in the sound frequency. The MFCC method is widely used for extracting speech recognition features and is regarded as a popular feature extraction method in speech-based biometric systems [26]. Recently, MFCC has been applied to identifying and classifying EEG signals [27–29].

Research found that humans have a different hearing sensitivity at different frequencies for sound waves. Here are some demonstrations about how it works. The frequency, *f*, of each tone is measured in Hz and the pitch is measured in the ‘Mel’ scale, as shown in Eq. (1) [30].

1 $$\begin{aligned} f_{mel}=2595log_{10}(1+\frac{f}{700}), \end{aligned}$$

where $f_{mel}$ is the pitch in Mels.

**Fig. 1:**
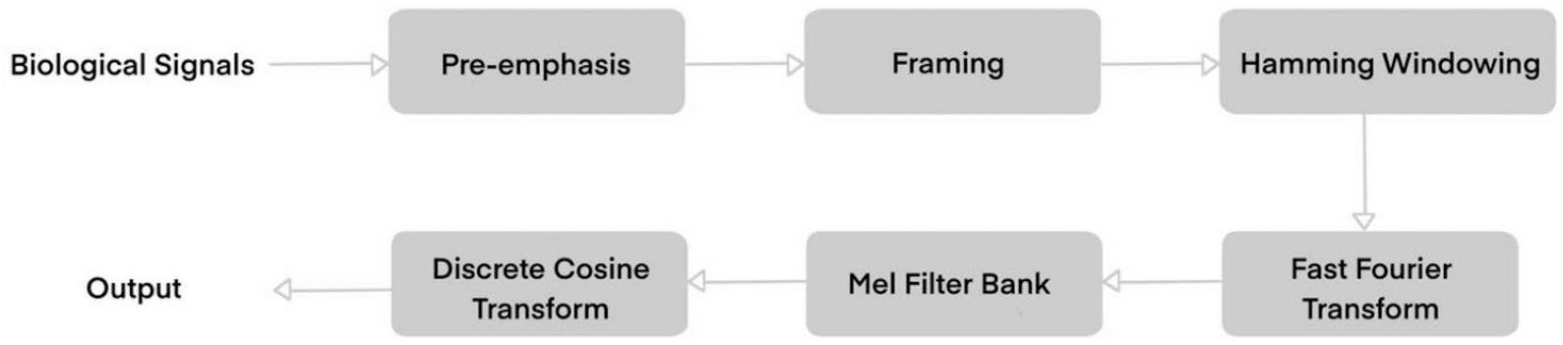
The flowchart of the MFCC features’ extraction processing

Figure 1 shows the overall flowchart of the MFCC feature extraction, which contains six steps:

Step 1: Pre-emphasis

The purpose of the pre-emphasis step is to enhance high-frequency signal segments. This step can lose energy easily during signal transmission.

2 $$\begin{aligned} y(n)=x(n)-a*x(n-1), \end{aligned}$$

here *n* represents the number of samples in each frame and *a* represents the parameter of pre-emphasis, *x*(*n*) is the input signal, and *y*(*n*) is the output signal. We consider $a = 0.97$ in our experiments, as 97% of any one sample is assumed to be the original signal from the previous signal.

Step 2: Framing

After the pre-emphasis step, each variable-length signal is separated into fixed-length segments, and this step is called framing. Generally, the framing step processes data samples into small frames with a length of 0.1 to 3 seconds (s). To avoid an omitting the signal by the window boundary, there must be a section of the overlap between each connected frame when offsetting a frame. The fixed-length segments are called frames. This is done to prevent characteristics from changing from one frame to another frame. In this paper, signal samples are divided into frames with a duration of 0.5 s, and its stride is 0.25 s.

Step 3: Hamming Windowing

Biological signals are constantly changing. It means that both ends of segmented frames lead to deviation. To eliminate the signal inconsistency, each frame is substituted into the Hamming windowing function which is defined as

3 $$\begin{aligned} w(n)=0.54-0.46\cos (\frac{2\pi n}{T-1}), \end{aligned}$$

where $0\le n\le T-1$. *T* is the hamming window length and *w*(*n*) represents the Hamming window. The coefficient values of 0.54 and 0.46 are generally the empirical values in our experiments. They are selected based on the specific situations.

Step 4: Fast Fourier Transform (FFT)

The output from the framing step is in time domain. To convert a time domain signal into a frequency domain one, the MFCC features are extracted through FFT. After the Hamming window has been applied, each frame is subjected to the FFT technique to obtain the energy distribution. The frequency spectrum of the frame is then obtained by performing the procedure on a windowed basis. The power spectrum of the sample is also obtained by taking the biological signal spectrum’s module square. The signal FFT is provided below in Eq. (4):

4 $$\begin{aligned} X_{a}(k)=\sum _{n=0}^{N-1}x(n)e^{-2\pi ikn/N}, \end{aligned}$$

where $0\le k\le N$ and *x*(*n*) represents the input signal. *N* is the data points while *a* indicates the input segment.

Step 5: Mel Filter Bank

The power spectrum of the samples is then obtained by following the FFT transformation step. During this process, the harmonics commonly utilized to enhance the original signal are removed. To minimize the amount of computation required, a Mel-scale triangular filter bank has been developed. This type of filter bank simulates the sound perception of a human ear by being more discriminative and less discriminative at lower frequencies.

**Fig. 2:**
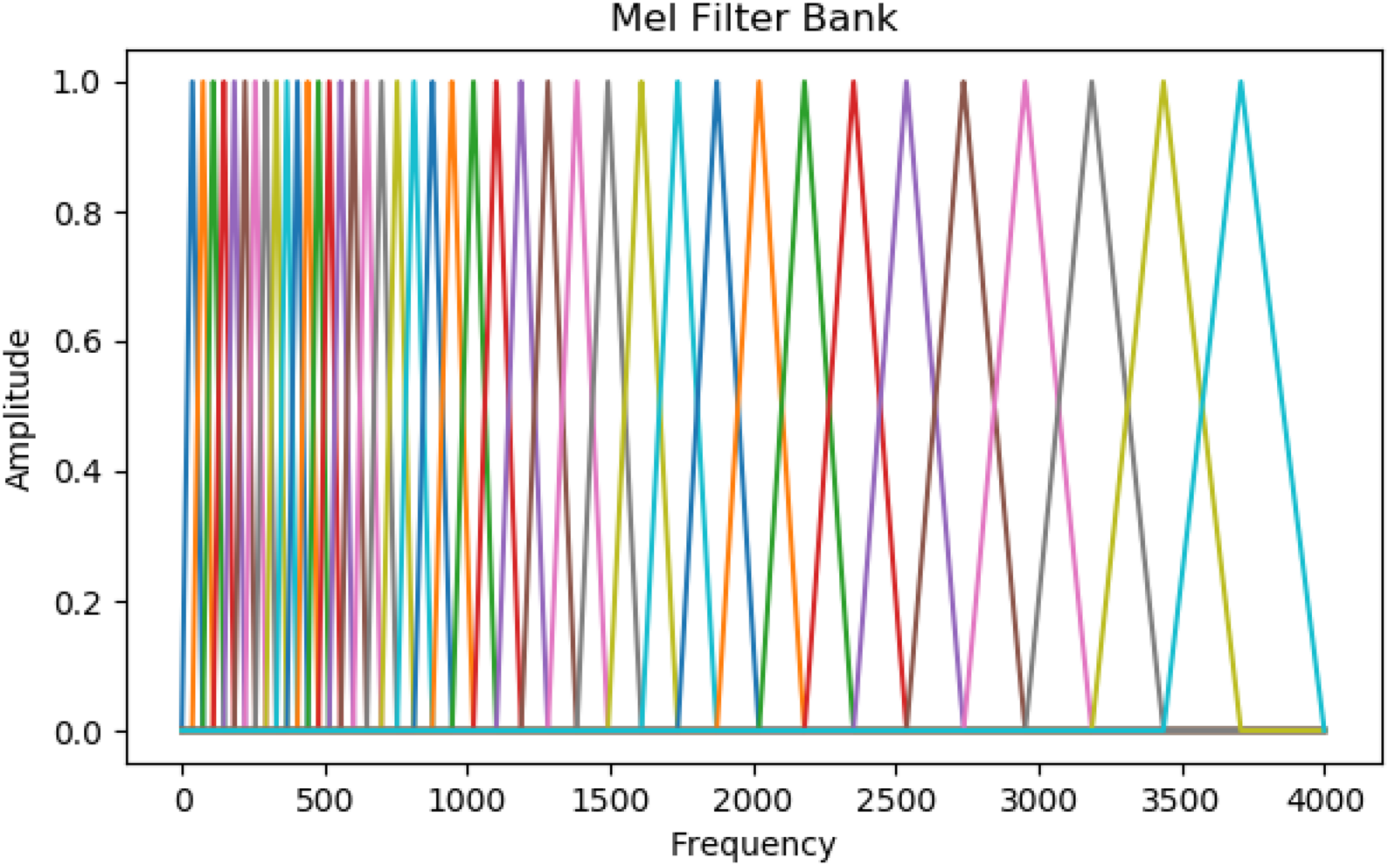
Mel-scale filter bank

A filter bank composed of *M* filters is similar to that of critical bands. Each of the 40 triangular filters in the bank is composed of a response at the centre frequency of 1, and its response decreases linearly as it reaches zero. Figure 2 shows the Mel-scale filter bank.

The filter response $H_{m}(k)$ of the *m*th filter in the Mel-scale filter bank is shown in Eq. (5):

5 $$\begin{aligned} H_{m}(k)={\left\{ \begin{array}{ll}0 &{} k< f(m-1)\\ \frac{k-f(m-1)}{f(m)-f(m-1)} &{} f(m-1) \le k< f(m) \\ 1 &{} k=f(m)\\ \frac{f(m+1)-k}{f(m+1)-f(m)} &{} f(m)< k\le f(m+1)\\ 0 &{} k > f(m+1)\end{array}\right. }. \end{aligned}$$

The log energy of each filter output is calculated. The outcome of log energies is defined in Eq. (6):

6 $$\begin{aligned} s(m)=\ln (\sum _{k=0}^{N-1}\left| X_{a}(k) \right| ^{2}H_{m}(k)), \end{aligned}$$

where $0\le m\le M$. *M* is the number of filters in the Mel-scale filter bank.

Step 6: Discrete Cosine Transform (DCT)

The filter bank coefficients computed in the previous step are highly correlated and can cause problems in a model learning method. Therefore, a discrete cosine transform (DCT) is applied to decorrelate the filter bank coefficients and produce a more compressed representation for the filter bank. The DCT is a variant of the Fourier transform (FT). The advantage of the DCT is that the results are the actual numbers with no imaginary parts. As mentioned above, 40 triangles are used in our experiments. In practice, only the first 12–20 points are collected and further compress the data. This step can convert the log Mel spectrum to the time series using the DCT. The outcome of the transformation is called the MFCC.

Finally, the DCT is applied to filter the bank energies to decorrelate the energies and the MFCC *C*(*n*) is defined below in Eq. (7):

7 $$\begin{aligned} C(n)=\sum _{m=0}^{N-1}s(m)\cos (\frac{\pi n(m-0.5)}{M}), \end{aligned}$$

where $n=1,2,...,L$ with *L* represents the desired number of MFCCs. *M* is the number of filters.

### Convolutional neural networks (CNNs)

In this section, a structure of a deep learning model is presented. This model can extract deep hidden features from a frequency domain and uses the calculated MFCC features for efficient sleep stage classification. A CNN combined with an LSTM layer is used. A CNN is a neural network that aims to process data with a grid-like structure [31]. The advantages of using a CNN are the features of parameter sharing, equivariant representations, and sparse interactions. Generally, a CNN model contains one input layer, multiple middle hidden layers, and an output layer. The multiple middle hidden layers are usually made by multiple convolutional layers, multiple pooling layers, a fully connected layer, and a softmax function. Normally any convolutional layer can play an essential role in the whole model. The learnable kernels in a convolutional layer can compute the feature maps and produce a new two-dimensional (2D) map.

In this paper, the first convolutional unit uses a 6$\times$6 kernel with a one-by-one data stream as the input (the segment signal details are provided in Section 2.6). The following three convolutional kernels are 5$\times$5, 5$\times$5, and 2$\times$2, respectively. Each convolutional layer is with a leaky rectified linear unit (LReLU) [32], with a negative slope of 0.1. After the convolutional layers, we present an LSTM layer, a fully connected layer, and a softmax activation function as a classification function for sleep stages.

**Fig. 3:**
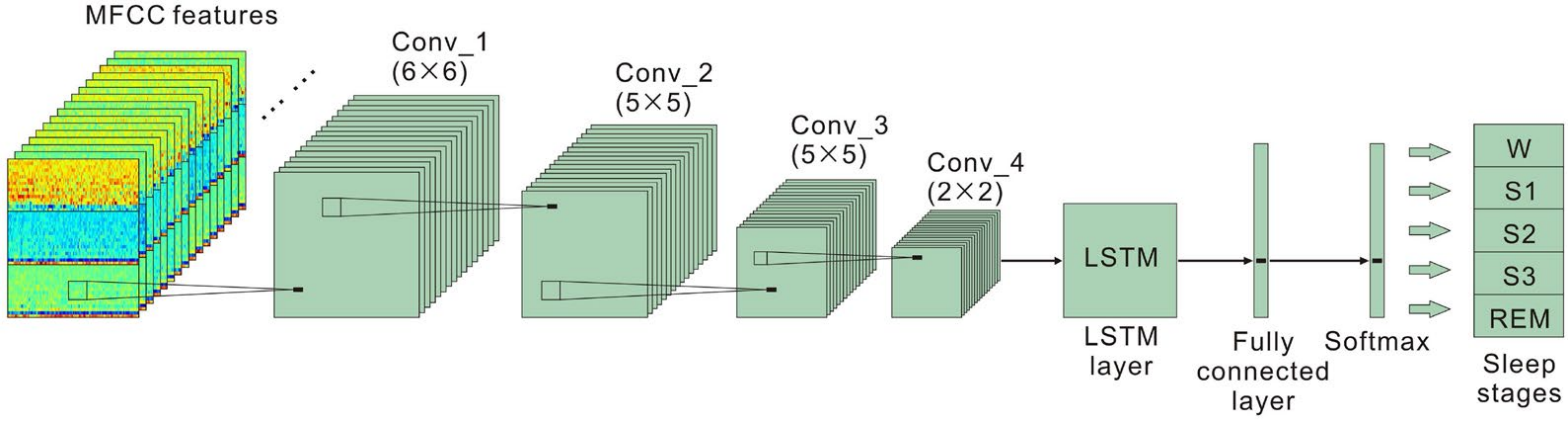
The architecture of the deep learning model

### Long short-term memory (LSTM)

This study combines the CNN with an LSTM as the deep learning model for classifying sleep stages. The architecture of the LSTM consists of memory function cells. Each cell utilizes a memory unit to manage the information in the network [33]. Each memory unit includes three gates, namely an input gate, an output gate, and a forget gate. Each of the three gates functions differently in the network with the input gate and the output gate manages the data flow of the network input and output, respectively. The forget gate controls the memory re-settings [34]. The LSTM network is designed to decrease the impact of the gradient problem. Some LSTM-based methods perform better for time series signals (sleep stage classification, natural language processing, speech recognition, and so on) than many machine learning methods [35] because an LSTM network architecture is a chained structure and better suit for time series data.

**Fig. 4:**
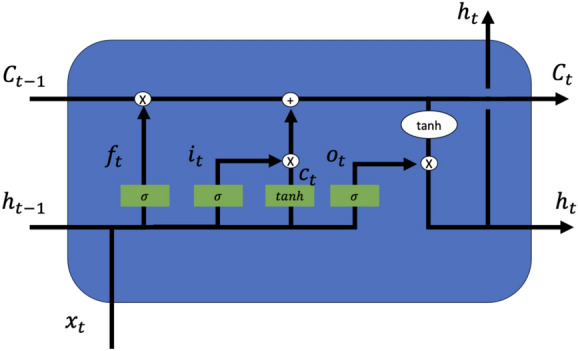
The structure of the LSTM cell

Each function in the main structure of the LSTM network is defined as below:

8 $$\begin{aligned} i_{t}= \,& {} \sigma (W_{xi}x_{t}+W_{hi}h_{t-1}+W_{ci}c_{t-1}+b_{i}) \end{aligned}$$

9 $$\begin{aligned} f_{t}= \,& {} \sigma (W_{xf}x_{t}+W_{hf}h_{t-1}+W_{cf}c_{t-1}+b_{f}) \end{aligned}$$

10 $$\begin{aligned} o_{t}= \,& {} \sigma (W_{xo}x_{t}+W_{ho}h_{t-1}+W_{co}c_{t}+b_{o}) \end{aligned}$$

11 $$\begin{aligned} c_{t}= \,& {} \tanh (W_{xc}x_{t}+W_{hc}h_{t-1}+b_{c}) \end{aligned}$$

12 $$\begin{aligned} C_{t}= \,& {} f_{t}C_{t-1}+i_{t}c_{t} \end{aligned}$$

13 $$\begin{aligned} h_{t}= \,& {} o_{t}\tanh (c_{t}) \end{aligned}$$

where $i_{t}$, $f_{t}$, $o_{t}$, $c_{t}$, $C_{t}$ and $h_{t}$ represent the input vector, forget input, output vector, cell input, cell output, and hidden layer output, respectively. $W_{x*}$ is the input weight, $W_{h*}$ represents the hidden layer weight, and $W_{c*}$ is peephole weight. The $\sigma ()$ is the sigmoid function. The structure of LSTM is shown in Fig. 4.

The deep learning architecture is shown in Fig. 3 and consists of four sections: four convolutional layers, one LSTM layer, a fully connected layer, and a softmax layer. In this study, the 128 output units from the LSTM layer are connected to a fully connected layer and then to a softmax layer, which then classifies the five sleep stages. The various models in this architecture are validated and tested to find the optimal network structure for deep learning. Five different network parameters are used in the study, such as the number of input epochs (1–4), the number of LSTM layers (1–5), and the number of LSTM units (16, 32, 64, and 128). The parameters were selected for their combination during the validation and training phase.

In this paper, the features of the MFCC framework are used as the model inputs to perform a one-dimensional signal recognition function. The final sleep stage classification model is provided in Fig. 3.

### Evaluation metrics

Five different evaluation measures are used to evaluate the performance of the proposed method, including the confusion matrix, accuracy (Acc), precision (Pre), Cohen’s kappa ($\kappa$), recall (Rec), and F1-score. These matrices are defined as follows:

14 $$\begin{aligned} Accuracy= & {} \frac{TP+TN}{TP+FN+FP+TN}\% \end{aligned}$$

15 $$\begin{aligned} Precision= & {} \frac{TP}{TP+FP}\% \end{aligned}$$

16 $$\begin{aligned} \kappa= & {} \frac{p_{0}-p_{e}}{1-p_{e}} \end{aligned}$$

17 $$\begin{aligned} Recall= & {} \frac{TP}{TP+FN}\% \end{aligned}$$

18 $$\begin{aligned} F1{\text {-}}score= & {} 2\frac{Precision\times Recall}{Precision+Recall} \end{aligned}$$

where TN, FN, FP, and TP are true negatives, false negatives, false positives, and true positives, respectively. TN represents the number of sleep stages wrongly classified as corresponding to the labeled sleep stages. FN denotes the number of sleep stages wrongly classified as the sleep stages should have been. FP is the number of sleep stages wrongly classified as labeled. TP means the number of sleep stages classified rightly labeled. The Cohen’s kappa coefficient is considered another performance evaluation measure in this study. It is used as a measure of agreement between the same data on a different method. It is generally considered to be more robust than percentage agreement [36].

This study used the Test Cost Index (TCI) to evaluate the model’s performance. The mathematical formula of TCI is provided below:

19 $$\begin{aligned} Test\ cost\ index\ = \frac{1}{N}\displaystyle \sum \limits _{i=1}^N(y_{i}-h_{\theta }(x_{i}))^3, \end{aligned}$$

where $y_{i}$ is true label of data, $h_{\theta }(x_{i})=\theta _{0}+\theta _{1}x$ ($x_{i}(i\in N)$ represents a feature vector), and $h_{\theta }$ $(x_{i})$ is predicted label.

### Pre-processing

**Fig. 5:**
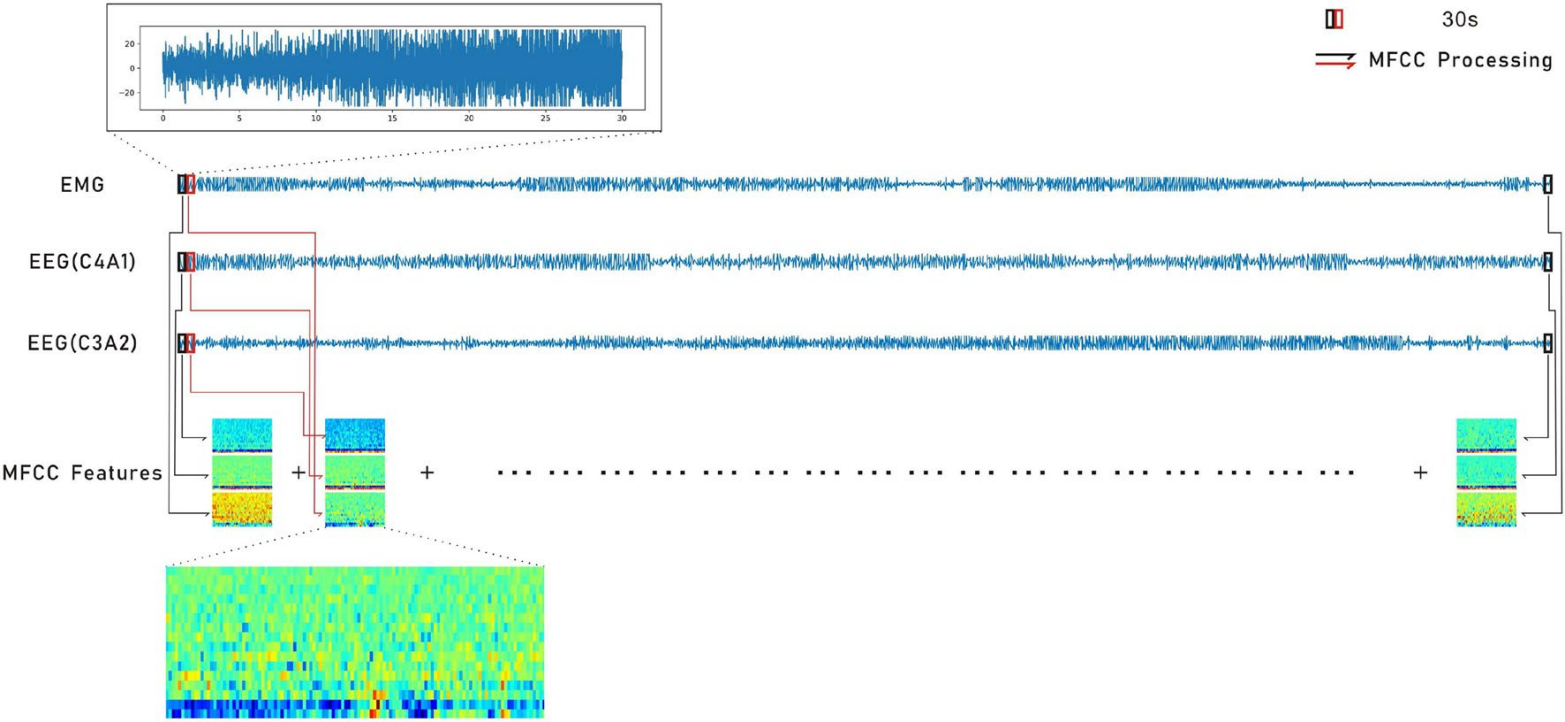
The process of MFCC feature extraction from multi-channel signals

The input into a convolutional layer is from three epochs, which is the input data stream from the MFCC features of the EEG (C3A2 and C4A1) and the EMG channel. This study was inspired by the work of Pei et al. [17] and by their excellent performance of a deep learning model using PSG multi-channel signals. This plan aims to extract more meaningful features from multi-channel PSG signals, which are used to classify sleep stages. The more the data streams that are used, the faster the deep learning model can learn how to improve its efficiency. For instance, by taking advantage of the multiple epochs of data, the features of the MFCC can be put in one frame. Two datasets, UDDB and SHHS, are used in this experiment. There are five independent channel signals, including two EEGs and one EMG. All the features of the MFCC are combined simultaneously as the new input data stream. Figure 5 shows the flowchart that describes how the new data stream is formed.

## Results and discussions

This section presents the experimental results by the proposed method for five sleep stage classification on the SHHS dataset and UCDDB dataset. The classification by the deep learning model is carried out in Pycharm (version Professional, 2020.3) [37] environment in a computer with a graphic card RTX 2080Ti and an Intel Core i9. The same computer performs all other mathematical calculations and data pre-processing in Pycharm. The proposed method is implemented in a Tensorflow framework [38]. The parameters of the MFCC method are first selected carefully by an empirical evaluation as the final sleep stage classification performance depends on the MFCC features. The MFCC method is repeated four times using the selected parameters for the same dataset to achieve a consistent and reliable outcome. The performance of the proposed method is also compared with other state-of-the-art methods.

### Parameter Selection for the MFCC features

This study employs the MFCC feature extraction method for sleep stage classification. The MFCC method has two key parameters, window and stride, $\{w, s\}$, respectively. Both $\{w, s\}$ should be carefully selected for an optimal performance as these two parameters have a high impact on the results. The window size parameter *w* determines the Fourier transform window size. The parameter *s* is the stride of the window movement, and it decides the size of the input data stream. The set of values of $\{w, s\}$ may be less a feature extraction matter rather a significant calculation efficiency issue. Regardless the parameters should be appropriately selected.

As introduced in Sect. 2, the definition and functions of each part for the MFCC method have been briefly described. Here, we present the results of several values of *w* and *s*. This study conducts several experiments using different *w* and *s* values for finding out optimal results for the sleep stage classification. A set of values of $\{w, s\}$ of 0.05 s, 0.1 s, 0.25 s, 0.5 s, 1 s, 1.5 s, 2 s, 2.5 s, and 3 s. There are 23 combination values of $\{w, s\}$, as shown in Table 2, and each set is tested three times.

**Table 2:** The results of the MFCC features based on different window and stride selection

Window (s)	Stride(s)									
	0.05		0.1		0.25		0.5		1	
	Acc (%)	Time(s)	Acc (%)	Time (s)	Acc (%)	Time (s)	Acc (%)	Time (s)	Acc (%)	Time (s)
0.10	69.69	1913	–	–	–	–	–	–	–	–
0.25	–	–	76.05	981	–	–	–	–	–	–
0.50	–	–	78.01	953	82.35	401	–	–	–	–
1.00	–	–	78.28	936	73.95	397	76.07	227	–	–
1.50	–	–	75.34	916	78.29	397	74.32	225	72.18	146
2.00	–	–	75.92	916	74.90	394	73.84	227	76.49	140
2.50	–	–	74.89	893	76.13	395	75.17	223	73.65	142
3.00	–	–	79.02	872	77.42	393	72.20	221	73.76	137

**Fig. 6:**
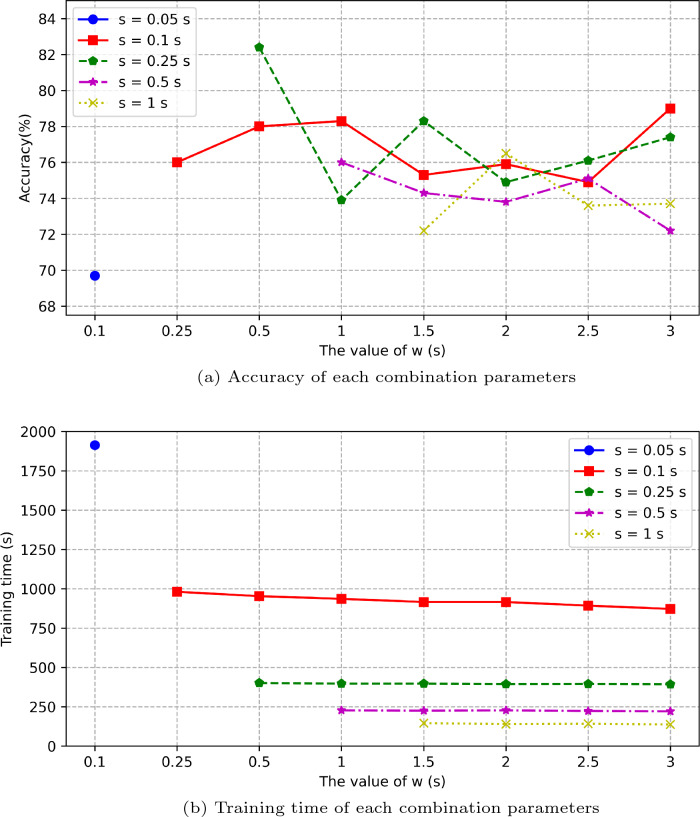
The classification accuracy and training time performance for different combinations of parameters (*w* and *s*) (The panel presents the stride values were 0.05 s, 0.1 s, 0.25 s, 0.5 s, and 1 s variation patterns)

Table 2 presents several combinations of $\{w, s\}$ value sets and Fig. 6 provides their performances, in terms of the accuracy and training time. Table 2 and Fig. 6 show that the training time has a strong connection with the *s* value. The training time is dramatically decreased when the *s* value has been increased. The accuracy is much more stable when *s*=0.1 s compared to *s*=0.25 s, 0.5 s, or 1 s. The experimental results show that the best performance is obtained when *w*=0.5 s and *s*=0.25 s. These values can provide the best results in terms of the balance of the accuracy and training time. When *s*=0.5 s and 1 s, the training time is reduced compared to *s*=0.25 s, but the accuracy results are decreased. In this study, the accuracy and training time as the model performance indicators are the critical factors in evaluating the model performance on the sleep stage classification.

Table 2 and Fig. 6 show that when *w*=0.5 s and *s*=0.25 s is the best combination for the MFCC feature extraction parameters for this proposed deep learning classification approach. These combination values are used in this paper.

### Experimental classification results

Table 3 shows the experimental results for the SHHS-100 dataset and the UCDDB dataset in terms of F1-score (%) for each sleep stage, test cost index (TCI), Cohen’s kappa ($\kappa$), and accuracy (ACC). In this study, the input data are divided as training, validation, and testing sets with a distribution rate of 70%, 20%, and 10%, respectively. All training data, validation data, and testing data streams are gone through the MFCC extraction and pre-processing phases before being input to the proposed deep learning model. Different numbers of subjects from the SHHS1 dataset and UCDDB dataset are used to train, validate, and test the deep learning architecture.

**Table 3:** Performance of the proposed scheme for two databases: UCDDB and SHHS1

Dataset	Number of Testing Epochs	Overall Metrics			F1-Score(F1) (%)				
		ACC (%)	$\kappa$	TCI	W	S1	S2	S3	REM
UCDDB	2560	73.07	0.63	1.45	78.14	78.98	52.75	75.67	67.41
SHHS1-100	10240	82.35	0.75	0.55	93.79	27.09	79.45	64.09	82.17

Table 3 shows that the SHHS1-100 in the proposed deep learning model yields the best sleep stage classification performance on the ACC, $\kappa$, TCI, and F1-score. The SHHS1-100 dataset [24], the 100 subjects as input, could achieve optimal results. The UCDDB dataset [25] has 25 subjects with an accuracy of 73.07% and fewer subjects input than the SHHS1-100 dataset. This could be the reason for unexpected results. The S1 stage in the SHHS1-100 dataset presents the lowest F1 score of 27.09%, as the S1 stage in the SHHS1 dataset has fewer epochs for the model training work. The W stage in SHHS dataset reports the highest result, as well as in the UCDDB dataset.

**Fig. 7:**
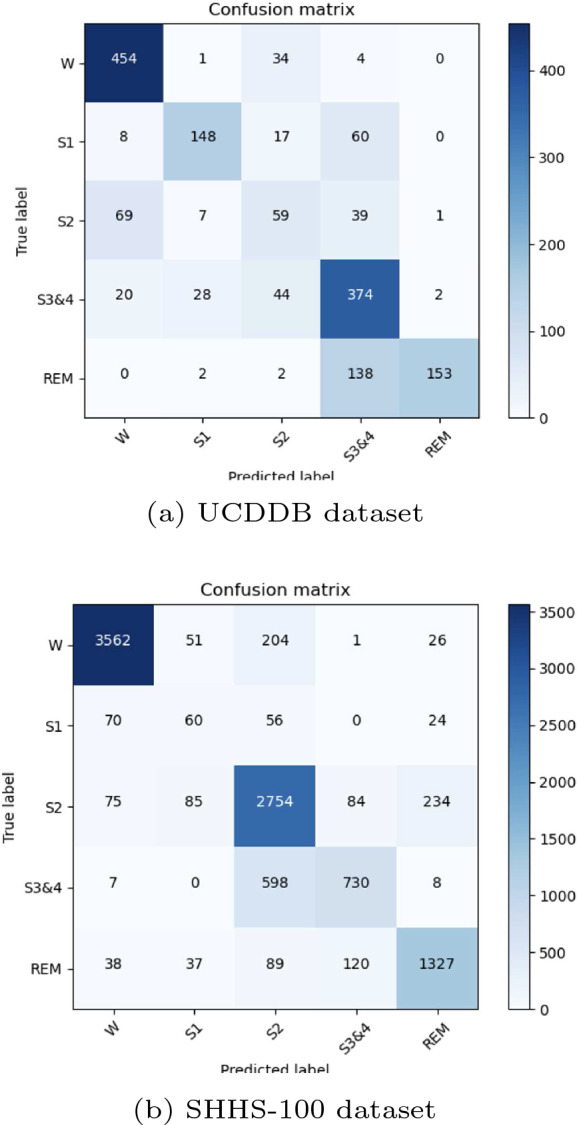
UCDDB and SHHS1-100 confusion matrix (The right side panel represents the number of predicted labels. The deeper color means more predicted labels)

Figures 7 provide the confusion matrix for two datasets.

To improve performance, several network structures and parameters’ settings for the proposed deep learning model have been tested. Firstly, three to six convolutional layers with a batch size of 64, 128, 256, and 512; a stride size of 2–4; and the filter size of 3, 5, 7, and 9 are tested separately. In the study, the output accuracy of the model was significantly improved when the parameters were chosen in Fig. 3.

**Fig. 8:**
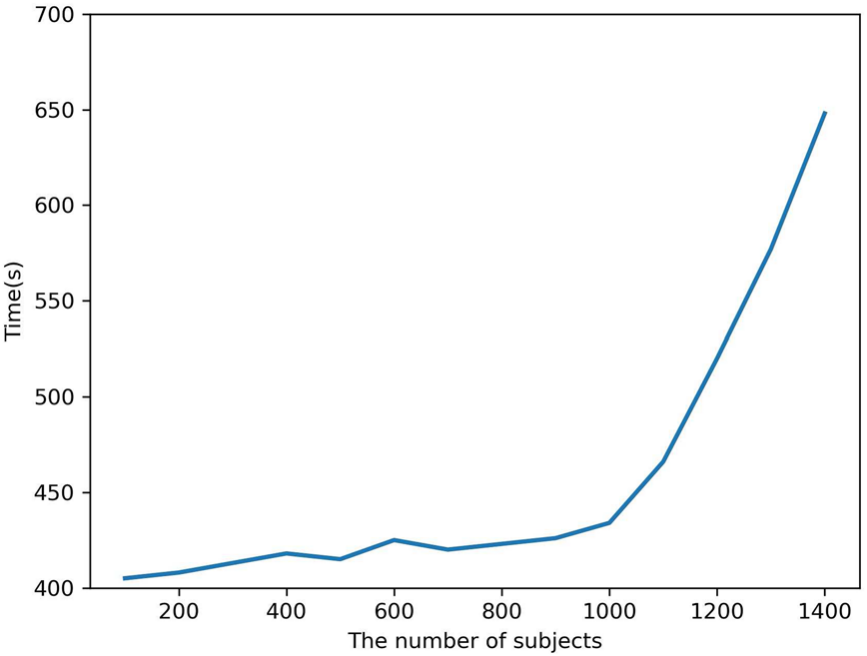
The execution time for the proposed method with different number of subjects

**Fig. 9:**
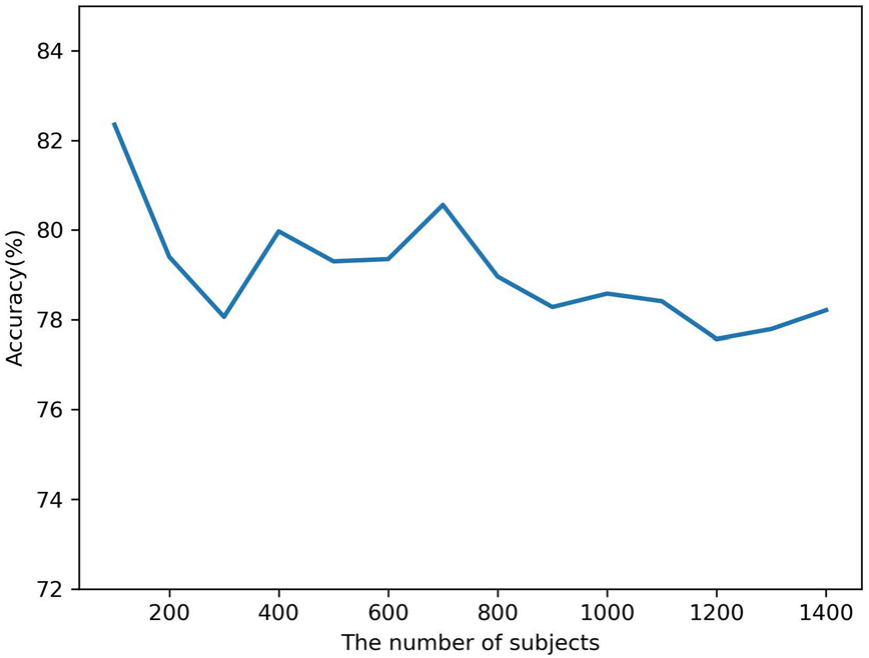
The performances of different number of subjects

To test the stability of the model and the impact of the different number of unhealthy subjects on its performance, we tested several experiments. Figures 8 and 9 show the execution time and accuracy of the proposed method. They represent the relationship between execution time and accuracy in two different datasets. The execution time of the proposed method mainly refers to the training and testing time. In each experiment, the data were run on a single model architecture and tested three times. The accuracy and efficiency of the proposed method are shown in Figs. 8 and 9. When the number of subjects is 100, the model achieves the highest accuracy and the lowest execution time. The execution time of the model significantly increases when the number of subjects exceeds 1000. The Cohen’s kappa and F1 scores have opposite decreases trend, as shown in Fig. 8. In Fig. 9, the accuracy of the dataset is also shown, which suggests a declining trend when the number of subjects exceeds 100. Its imbalance distribution of the sleep stages can be explained and could be found in the SHHS1 database.

### Discussions

This study focuses on the utilization of a lightweight deep-learning model on the sleep dataset to accurately categorize different sleep stages. This study introduced a deep learning model that integrates a CNN with an LSTM model. The proposed approach can extract features from the frequency domain. The MFCC is derived from EMG and EEG data and then inputted into convolutional layers. The obtained representations are fed into an FC layer and a softmax classifier to carry out sleep scoring. The findings suggest that the proposed approach achieves an acceptable performance in sleep scoring tasks by employing frequency domain features retrieved from the raw data. An advantage of using this feature is its capacity to support the input of MFCC features, which are a 2D data stream. This allows for the preservation of detailed information about each sleep stage. Furthermore, the use of 2D data streams has the capacity to improve the effectiveness of the training process and simplify the extraction of important information from the 1D stream. Another benefit of this approach is its capacity to reduce the requirement for intricate architecture, thereby improving computational efficiency. Utilizing previous research, we have implemented a comparable structural model [17] to categorize sleep stages using identical datasets. It employed a 1D signal as its input and exhibited good performance. However, the computational efficiency of the suggested model exceeds that of the original study when trained on the same hardware. Using the MFCC features contributes to significant computing power. Furthermore, the outcomes demonstrate exceptional efficiencies and substantially decrease the time required for execution, which was regarded as a notable enhancement in comparison to several existing approaches in Table 4. Moreover, employing MFCC as input has the potential to enhance the results by eliminating noise during the extraction process. The generality and efficiency of the proposed model are praiseworthy. The results suggest that the utilization of MFCC features can effectively forecast the various sleep stages.

**Table 4:** Comparison between our experiment and other sleep stage classification methods

Article	Dataset	Method	Channel	Subjects	ACC (%)	$\kappa$	F1-score				
							W	S1	S2	S3	REM
Phan et al. [15]	Sleep-EDF	Multitask 1-max CNN	Fpz-Cz	20	81.9	0.74	–	–	–	–	–
Qu et al. [20]	Sleep-EDF	CNN	Fpz-Cz	20	84.3	0.78	90.2	48.3	87.8	85.6	83.0
Supratak et al. [38]	Sleep-EDF	DeepSleep- Net	Fpz-Cz	20	82.0	0.76	84.7	46.6	85.9	84.8	82.4
Sors et al. 39	SHHS1	CNN	C4-A1	5728	86.8	0.81	91.4	42.7	88.0	84.9	85.4
Seo et al. 40	SHHS1	IITNet	C4-A1	5728	83.6	0.77	88.7	21.3	86.1	84.9	78.1
Eldele et al. 41	SHHS1	AttnSleep	C4-A1	329	84.2	0.78	86.7	33.2	87.1	87.1	82.1
This study	SHHS1	CNN+LSTM	C4-A1, C3-A2, EMG	100	82.4	0.75	93.8	27.1	79.5	64.1	82.2

Table 4 presents a comparative report for the proposed method with some existing state-of-the-art studies for the sleep stage classification by deep learning based methods. The proposed method produced an accuracy of 82.35% and Cohen’s kappa of 0.75. From Table 4, Phan et al. [15] and Qu et al. [20] designed a model based on a CNN network and used the same Sleep-EDF dataset with good results. Phan et al. [15] reported an accuracy of 81.9% and a Cohen’s kappa of 0.74, and Qu et al. [16] reported an accuracy of 84.3% and a Cohen’s kappa of 0.78, respectively. Supratak et al. [39] proposed a DeepSleepNet method based on a CNN and a bidirectional LSTM. They showed an accuracy of 82% and a Cohen’s kappa of 0.76. The methods in [15, 20] and [39] were also based on a CNN model and classified the Sleep-EDF dataset. They reported comparable results that were similar to the ones presented in this paper. However, [15, 20] and [39] all used only 20 subjects and all those subjects are healthy.

The SHHS dataset [24] includes subjects with sleep disorders, breathing problems, and other healthy issues that could influence on sleep stage classification performance. Qu et al. [20] reported that it took 2.5 hours (h) to train their model with all 20 subjects, while the proposed model in this study only needs around 400 s for the model training and testing for 100 subjects. The computation efficiency of the proposed model is one of the main advantages of this paper. Furthermore, it also achieves a satisfying performance compared to other studies on sleep stage classification [40–42]. Sors et al. [40] designed a deep CNN model for automatic sleep scoring on the entire SHHS1 dataset and used four connected epochs as input with an accuracy of 86.8% and a Cohen’s kappa of 0.81. Seo et al. [41] proposed an IITNet method that utilized the residual neural network and bidirectional LSTM and reported the results with an accuracy of 83.6% and a Cohen’s kappa of 0.77. Eldele et al. [42] developed an AttnSleep method for automatic sleep stage classification. AttnSleep is based on multi-resolution CNN and adaptive feature recalibration (AFR). Eldele et al. [42] selected 329 subjects in the SHHS1 dataset for training and testing. They reported an accuracy of 84.2% and a Cohen’s kappa of 0.78. According to the researchers [40–42], their methods achieved a good performance on the SHHS1 dataset. The results in this paper prove that the proposed model produces a better classification performance for more subjects. While the methods [40–42] reported approximately 2 days, 10 h, and 2.1 h on the model computational time, respectively. Compared to those methods [40–42], the proposed method significantly improved the model execution time.

Table 4 shows the state-of-the-art sleep stage classification methods reported in the literature, and most of them are based on a CNN architecture. The methods in [15, 20, 39] used a single EEG signal for sleep stage classification, and all chose 20 subjects from the Sleep-EDF dataset. Our proposed method has a similar model performance to [15, 20, 39] but using more subject data and with unhealthy subjects. For our testing object, the number of sleep disordered subjects, such as breathing and heart problems, would impact on a model performance. In addition, [39–41] and our study all chose the SHHS1 dataset as the testing subject, all with acceptable model output. On the model output performance, this proposed model for the first uses the MFCC features extracted from bio-signal for the sleep stage classification and achieves a satisfied result. However, there are potential issues that need to improve in our study.

Table 4 and Fig. 8 present the execution time of 100 subjects to 1400 subjects by the proposed model. It can be noticed that its computation time is much less time than those by methods in [40–42]. This is the main advantage of this study compared to other state-of-the-art methods.

## Conclusion

Human sleep research aims to explore effective feature extraction that reaches a higher accuracy to the limit of sleep staging. They also expect a reliable method which is automatically and efficiently classifies sleep stages. Hence, a deep learning method is designed for sleep stage classification using the MFCC features extracted from bio-signals. The proposed model is based on a CNN and an LSTM. It uses convolutional layers to extract the MFCC feature, which is an effective feature for the sleep stage classification and extract from multi-channel bio-signals. Thus, the MFCC feature and the neural network learning are performed to the attention of more useful features from raw signals. This also reduces the impact of the noise in the original signal on the results. Finally, an LSTM layer has been used to automatic learning the transition rules. The results show that the MFCC feature and the proposed method effectively classify the sleep stages. However, the MFCC feature extraction can negatively affect the results when the combination of the window size and the stride value is insufficient to extract the useful feature from the bio-signals. This study has explored the impact of the window size and the stride value on the experimental performance. The overall performance was acceptable when the window size was 0.5 s and the stride value was 0.25 s. Moreover, the experiment results show that Cohen’s kappa and the accuracy are 0.75 and 82.35%, respectively, on the SHHS dataset. It also presents Cohen’s kappa and accuracy as 0.63 and 73.07%, respectively, on the UCDDB dataset. It could prove the MFCC features from multi-channel bio-signals and the proposed method is efficient in the sleep stage classification area. On the other hand, the proposed deep learning model can calculate more efficiency than some existing state-of-the-art methods. The fewer model layers and extracted features from the raw signal support higher computing efficiency than other existing methods. Fortunately, our proposed method achieved good calculation efficiency in sleep stage classification by the deep learning method. It could prove the model has practical meaning in sleep stage classification and still has potential limitations. In the future, we plan to design a deep learning model based on state-of-the-art technics and networks on sleep stage classification. It can illustrate huge data subjects and still retain the calculating efficiency power.

## Data Availability

The datasets generated during and/or analyzed during the current study are available from the corresponding author on reasonable request.
